# Screening of Two Neighboring *CFTR* Mutations in Iranian Infertile Men with Non-Obstructive Azoospermia

**DOI:** 10.22074/ijfs.2016.4593

**Published:** 2016-11-01

**Authors:** Somayeh Heidari, Zohreh Hojati, Majid Motovali-Bashi

**Affiliations:** Department of Biology, Faculty of Sciences, University of Isfahan, Isfahan, Iran

**Keywords:** *CFTR*, Mutation, Azoospermia, Male Infertility

## Abstract

The genetic association between cystic fibrosis transmembrane conductance regulator (*CFTR*) gene mutations and male infertility due to congenital bilateral absence of vas deferens (CBAVD) is well established. Mutant *CFTR*, however may also be involved in the etiology of male infertility in non-CBAVD cases. The present study was conducted to estimate the frequency of ∆I507 and ∆F508 *CFTR* gene mutations in Iranian infertile males. We undertook the first study of association between these *CFTR* mutations and non-obstructive azoospermia in Iran. In this case-control study, 100 fertile healthy fathers and 100 non-obstructive azoospermia’s men were recruited from Isfahan Infertility Center (IIC) and Sari Saint Mary’s Infertility Center, between 2008 and 2009. Screening of F508del and I507del mutations was carried out by the multiplex-ARMS-PCR. Significance of differences in mutation frequencies between the patient and control groups was assessed by Fisher’s exact test. The ΔF508 was detected in three patients. However there are no significant association was found between the presence of this mutated allele and infertility [OR=9.2 (allele-based) and 7.2 (individual-based), P=0.179]. None of the samples carried the ΔI507 mutation. Altogether, we show that neither ΔI507 nor ΔF508 is involved in this population of Iranian infertile males with non-obstructive azoospermia.

## Introduction

Reproductive failure is associated with various genetic disorders, mainly numerical and structural chromosome abnormalities and gene mutations. At the genic level, male infertility has been linked with protamine gene mutations (1,2), 5-alpha reductase deficiency (3), androgen receptor gene mutations (4,5) and cystic fibrosis transmembrane conductance regulator gene (*CFTR*) mutations (6,14). More than 1950 *CFTR* variants have been identified in different ethnic populations, as curated in the cystic fibrosis genetic analysis consortium database. Many of these mutations are associated with a wide spectrum of phenotypes, including respiratory distress, chronic pancreatitis and male infertility (11). Male infertility caused by congenital bilateral absence of vas deferens (CBAVD) has been reported in more than 95% of men with cystic fibrosis (CF) (7,10). The genetic association between *CFTR* mutations and male infertility due to CBAVD is well established (10,12,15). Several studies have revealed involvement of *CFTR* mutations in other forms of male infertility due to defective spermatogenesis (10,16,20). Although a two-five fold increased in *CFTR* mutation rate in males with non-obstructive azoospermia has been reported (REF), a number of reports did not find any association between them (10,17,20,23). van der Ven et al. (20) investigate the possible involvement of *CFTR* in the etiology of non-CBAVD male infertility. Semen specimens from 127 unrelated healthy males with various diagnoses of reduced sperm quality were screened for a panel of 13 *CFTR* mutations. Fourteen of 80 (17.5%) infertile men due to reduced sperm quality and 3 of 21 (14.3%) men with azoospermia had at least one *CFTR* mutation (one azoospermic male was a compound heterozygote). No mutations were found in the control group of 26 individuals with normal semen parameters. This overrepresentation of *CFTR* mutations in men with reduced sperm quality and in men with azoospermia without CBAVD suggests that *CFTR* protein may be involved in the process of spermatogenesis or sperm maturation apart from playing a critical role in the development of epididymal glands and the vas deferens (20). Schulz et al. (10) investigated the frequency of *CFTR* mutations in 597 males with reduced sperm quality and 34 (5.70%) carried a mutation, indicating a two-fold higher frequency than in the general population. Boucher et al. (22) screened 39 patients with azoospermia without CAVD and 37 patients with severe oligozoospermia for a panel of 10 *CFTR* mutations. None of the *CFTR* mutations were observed in the patient group and suggested that *CFTR* gene is not involved in spermatogenesis. 

Some studies have reported expression of the *CFTR* gene in human Sertoli cells, germ cells and testes, suggesting its possible involvement in spermatogenesis (24,26). Based on these investigations, to date, it remains uncertain whether screening of *CFTR* mutations should be recommended for infertile males with non-obstructive azoospermia during assisted reproduction technology. There are just a few studies reporting the association between *CFTR* mutations and non-obstrucive azoospermia, especially in the Iran. The aim of this study was to estimate the frequency of ∆I507 and ∆F508 *CFTR* mutations in Iranian infertile males with non-obstructive azoospermia. We undertook the first study of association between *CFTR* gene mutations and non-obstructive azoospermia in Iran. 

This study was a case-control study. Blood samples were collected from 100 males with nonobstructive azoospermia in the Isfahan Infertility Center (IIC) and Sari Saint Mary’s Infertility Center, Iran, between 2008 and 2009, and from 100 normal men (men with normal fertility with at least one child and normal sperm parameters). All the patients and control individuals gave informed written consent to be included in the study. The diagnosis of non-obstructive azoospermia was based on the following examinations: normal semen volume, normal testicular size, presence of the vas deferens by clinical examination, normal levels of serum follicle-stimulating hormone (FSH), azoospermia, absence or low levels of fructose and absence of spermatozoa in sample extracted by percutaneous testicular sperm aspiration (TESA). No symptoms of CF including chronic lung inflammation/infection, pancreatic insufficiency and intestinal obstruction were reported in the clinical files of the patients. The mean age of the patients and controls were 31.5 and 30 years, respectively. In this study, all patients were azoospermic with an absence of spermatozoa in the semen and sample extracted by TESA. 

The ΔF508 and ΔI507 mutations in exon 10 of *CFTR* were selected for screening. The specificity of the primers were analyzed using Oligo®7 software (Version 7.0, Rychlik, 2007) (27). Further comparison of designed primers with the exon 10 sequence of *CFTR* was performed by CLC software (www.clcbio.com/genomics). 

Two ml blood was collected from each patient and normal control in tubes containing EDTA. Leukocyte genomic DNA was extracted from blood samples using the standard method of salting out with slight modification (28). Genomics DNA samples were stored in -20˚C after determining their relevant concentrations and quality on gels. 

**Fig.1:**
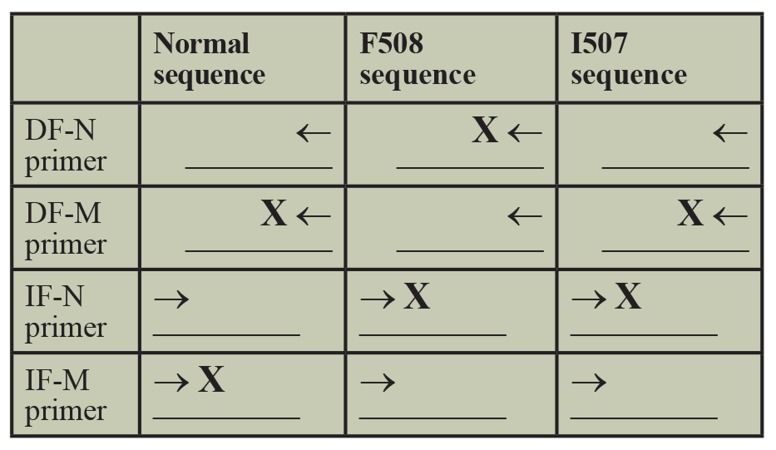
Visial representatin of wild-allele, ΔF508 and ΔI507 mutant ARMS primers for the amplification of the target sequence. The diagrams in the boxes align the normal and mutant ARMS primers (5' to 3') with the normal, ΔF508, and ΔI507 target DNA sequences.

Multiplex-ARMS-PCR was carried out in two separate reactions. This method is schematically drawn in the Figure 1. 

The genotype of an individual can be determined by analysing of the amplification products. Sequences of all primers were shown in Table 1.

**Table 1:** List of primer sequences


Primer name	Sequences ( 5ˊ to 3ˊ )

FC	GGT TTT ATT TCC AGA CTT CAC TTC ATA T
RC	TGC ATA ATC AAA AAG TTT TCA CAT AGT T
DFN	GTA TCT ATA TTC ATC ATA GGA AAC ACC ACA
DFM	GTA TCT ATA TTC ATC ATA GGA AAC ACC AAT
IFN	CTG GCA CCA TTA AAG AAA ATA TCA TCT T
IFM	CTG GCA CCA TTA AAG AAA ATA TCA TTG G


**Fig.2:**
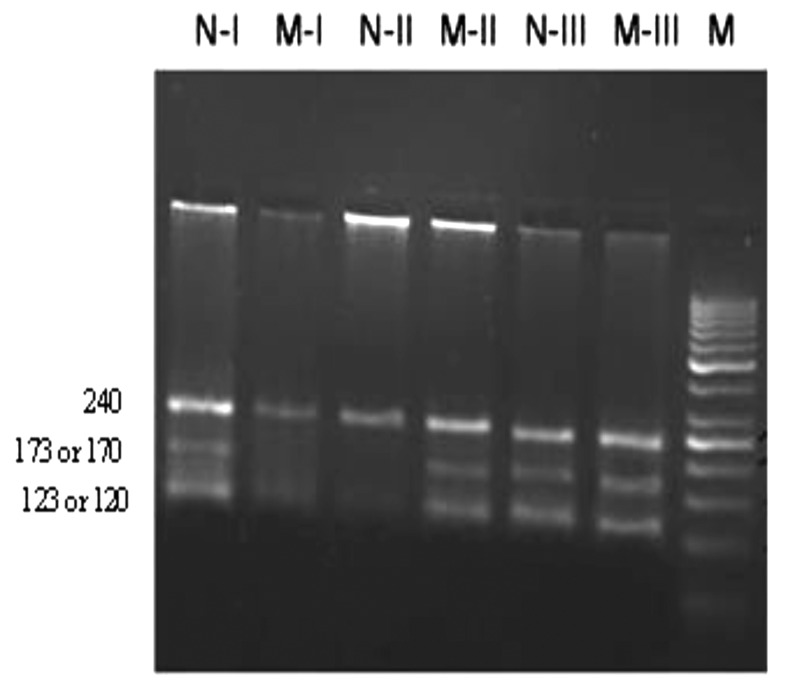
Detection of ΔF508 *CFTR* mutation in infertile men by using multiplex-ARMS-PCR. The amplification products of normal primers sets are shown here (N). M indicates the amplification products by IFM and DFM primers. The resulted amplified fragments for a normal and ΔF508 mutant are shown here. Sample I is a wild homozygote, sample II is a mutant homozygote (ΔF508) and sample III is a heterozygote (ΔF508). Fragment sizes are in the base pairs (bp). Marker; 50 bp DNA ladder. N-I; Normal primer sets for sample I, M-I; Mutant primer sets for sample I, N-II; Normal primer sets for sample II, M-II; Mutant primer sets for sample II, N-III; Normal primer sets for sample III, and M-III; Mutant primer sets for sample.

Wild-allele specific primers (DFN and FC primers) and ΔF508 mutant-allele specific primers (DFM and FC primers) produce 173 bp and 170 bp fragments, respectively. Amplification of sequence by wild-allele specific primers (IFN and RC primers) and ΔI507 mutant-allele specific primers (IFM and RC) give 123 bp and 120 bp fragments, respectively (Fig .2).

DNA amplification was carried out in duplicate for all samples. Each 25 μl reaction mixture contained 3 μl template DNA (50-100 ng), 1.6 μl MgCl_2_ (2.0 mM), 2.5 μl 10X PCR buffer, 1.5 μl FC (20 pmol ml-¹), 1.5 μl RC (20 pmol ml-¹), 0.75 μl DFN (20 pmol ml-¹), 0.75 μl IFN (20 pmol ml-¹), 1 μl dNTP mix (10 mM), 0.4 μl Taq polymerase (5 U) and 12 μl ddH2O. The reaction mixtures were prepared and kept on ice until the heating block of the thermal cycler reached the denaturation temperature (94°C). The PCR amplification was carried out at 94°C for 10 minutes and then followed with 32 amplification cycles of 40 seconds at 94°C, 1 minute at 58.8°C, 1 minute at 72°C, and a final extension at 72°C for 10 minutes. Amplification products were separated by electrophoresis using a 2.5% Metaphor agarose gel, stained with ethidium bromide and visualized by ultraviolet illumination.

Significance of differences in mutation frequencies between the patient and control groups was assessed by Fisher’s exact test (SPSS software version 16.0) and P values<0.05 were considered statistically significant.

The percentage difference of ΔF508 mutation between the patient and control groups was although not statistically significant (P=0.179), possibly due to the relatively small sample size, it displayed a large effect size [OR=9.2 (allele-based) and 7.2 (individual-based)]. No individuals carried the ΔI507 mutation.

The genetic link between *CFTR* mutations and a genital form of male infertility (CBAVD) is well established. Thus, screening of *CFTR* mutations is proposed for all infertile men with CBAVD, however, the association of *CFTR* mutations and non-CBAVD male infertility, is uncertain. Recently Xu et al. (29) demonstrated *CFTR*-dependent regulation of CREB in human Sertoli cells which suggests that its defective regulation may cause spermatogenesis failure as seen in non-obstructive azoospermia. This seems to be the possible mechanism by which *CFTR* may be involved in spermatogenesis. There are a few studies which have specifically looked at the frequency and role of *CFTR* mutations in azoospermic men without CBAVD (20, 23, 30-32). Many reports to date have shown conflicting results. Some studies showed significant association between *CFTR* mutations and non-obstructive azoospermia while the other investigations ruled out this association (14). In the present study, ΔF508 mutation was observed twice in heterozygous form (2%) and once in homozygous form among the 100 non-obstructive patients. This result is consistent with previous studies. For instance, Safinejad et al. (6) evaluated five common *CFTR* mutations (ΔF508, G542X, R117H, W1282X and N1303K) in Iranian infertile men with non-CAVD obstructive azoospermia. The common *CFTR* mutations were found in 9.43% (5.53%) patients for ΔF508 mutation. Another study by Sharma et al. (11) analyzing the frequency of *CFTR* mutations in infertile Indian males with non-obstructive azoospermia (n=60) and spermatogenic failure (n=150), showed that ΔF508 mutation was observed in 3.6% of patients with non-obstructive azoospermia.

However, some reports revealed the absence of ΔF508 mutation among all infertile patients (7, 22, 33). Ravnik-Glavac et al. (23) screened 80 men with idiopathic azoospermia, 50 men with severe oligozoospermia, 70 men with oligoasthenoteratozoospermia, and 7 men with CBAVD as well as 95 controls from Slovenia for mutations in 10 *CFTR* exons where the majority of the common CF disease causing mutations have been detected. The frequencies of *CFTR* mutations did not differ significantly between the control group and men with idiopathic nonobstructive azoospermia and subfertility, suggesting that *CFTR* mutations are not associated with spermatogenic failure and non-obstructive pathology of urogenital tract in men.

Moreover, we did not observed the ΔI507 mutation among all infertile patients and control individuals suggesting its rarity as a cause. In conclusion, our results did not show any significant association between these two mutations and non-obstructive azoospermia.

The studied population is informative but its size is not large enough to make definitive inferences about the involvement of these two mutations in non-obstructive azoospermia.

Further studies with greater number of *CFTR* mutations and patients are therefore needed to examine the role of *CFTR* in non-obstructive azoospermia.
